# The Injection of Gels Through an Intact Annulus Maintains Biomechanical Performance without Extrusion Risk

**DOI:** 10.3390/gels10040269

**Published:** 2024-04-17

**Authors:** Hans-Joachim Wilke, Holger Fuchs, Karin Benz, Juergen Mollenhauer, Christoph Gaissmaier, Frank Heuer, Cornelia Neidlinger-Wilke

**Affiliations:** 1 Institute of Orthopaedic Research and Biomechanics, Centre for Trauma Research Ulm, Ulm University, 89081 Ulm, Germanycornelia.neidlinger-wilke@uni-ulm.de (C.N.-W.); 2 NMI Natural and Medical Sciences Institute, The University of Tübingen, 72770 Reutlingen, Germany; karin.benz@tetec-ag.de (K.B.);; 3 TETEC Tissue Engineering Technologies AG, 72770 Reutlingen, Germany; christoph.gaissmaier@tetec-ag.de

**Keywords:** disc degeneration, autologous-disc-derived chondrocyte transplantation, hydrogel carrier material, biomechanical testing, in vitro study, nucleus replacement, disc regeneration, chondrocyte transplantation, cell therapy

## Abstract

For autologous-disc-derived chondrocyte transplantation (ADCT) a transglutaminase crosslinked gelatine gel and an albumin hyaluronic acid gel, crosslinked with bis-thio-polyethylene glycol, were injected through a syringe into a degenerated intervertebral disc, where they solidified in situ. This biomechanical in vitro study with lumbar bovine motion segments evaluated disc height changes, motion characteristics in a quasi-static spine loading simulators, and the potential extrusion risk of these biomaterials in a complex dynamic multi-axial loading set-up with 100,000 loading cycles. After the injection and formation of the gel in the center of the nucleus, the disc height increase was about 0.3 mm. During cyclic testing, a gradual decrease in height could be detected due to viscoelastic effects and fluid loss. No gel extrusion could be observed for all specimens during the entire test procedure. A macroscopic inspection after dissections showed an accumulation of the solidified gel in the center of the nucleus. The results demonstrate that the injection of in situ solidifying gels through the intact annulus allows for the stable maintenance of the injected gel at the target location, with high potential for use as a suitable scaffold to anchor therapeutically applied cells for disc regeneration within the treated nucleus pulposus.

## 1. Introduction

Regenerative therapeutic approaches using autologous intervertebral disc (IVD) cells or stem cells have shown promising results and therefore attracted more and more interest in recent years [1,2,3,4,5,6,7,8,9,10,11,12]. Knowledge regarding regenerative treatment approaches for degenerative disc diseases has increased through the use of animal models [13]. The safe administration of therapeutic cells with long-term maintenance at the site of injection is essential for a successful cell therapy approach. In the past, studies involving the reinjection of autologous cells harvested from prolapsed discs reached clinical trials with only preliminary success as they did not technically fulfil the requirements for such procedures [6]. Although cell therapy has great potential for intervertebral disc regeneration, the overall strength of the evidence regarding its safety is rather poor due to the substantial risk of bias, small sample sizes, and lack of suitable controls in these studies [14]. To ensure the safe and sustainable performance of therapeutic cells, finding the correct application strategy is crucial. The injection of fluid suspensions is associated with the problem of immediate leakage at the injection site [15,16]. Moreover, it will take a long time for the injected cells to be able to produce enough matrix to restore the disc matrix that is lost due to herniation or degeneration [17]. Therefore, supporting therapeutic cells containing a suitable biomaterial carrier that mimics the biomechanical disc matrix properties is a more promising approach. 

Hydrogels have the advantage of having similar mechanical characteristics and water-binding properties as the natural disc matrix. They can immediately replace the disc matrix that was lost because of degenerative changes or disc prolaps. With their ability to compensate for the water-binding properties of the NP [18], hydrophilic injectable hydrogels are considered ideal candidates for minimally invasive NP regeneration. Combining them with cells is a promising strategy to support matrix formation and the maintenance of tissue homeostasis [19]. Natural hydrogels used for NP therapy include hyaluronic acid (HA), collagen type I or II, fibrin, gelatin, alginate, chitosan, and gellan gum [19,20]. A variety of synthetic hydrogels, such as polyethylene glycol, polyvinyl alcohol, polyvinyl-pyrollidone, polyurethan, and cellulose, have also been described for NP tissue engineering approaches [19,20,21]. More recent studies used hydrogel scaffolds as cell carriers for the regeneration of degenerated disc tissue [22]. With their hydrophilic properties, high water content, and suitable biocompatibility, hydrogels have similar properties to the natural extracellular matrix (ECM) and can therefore be used for the delivery of drugs and proteins, as well as serving as carriers for therapeutic cells [23]. As they are a natural component of the NP matrix, natural hyaluronan-based hydrogels have promising properties, supporting NP repair. These thermoreversible hydrogels have been shown to be suitable for supporting the phenotype of NP cells, as well as allowing for the appropriate differentiation of bone marrow stromal cells in vitro and in organ culture [24,25]. 

A previous study focused on a hydrogel composed of chemically activated albumin, crosslinked using bis-thio polyethylene glycol, with promising rheological properties. This could form a stable scaffold, which was could support functional intervertebral disc cells in vitro and in vivo within a physiologic environment [26]. In a porcine nucleotomy model, a short-term follow-up of a disc cell therapy using albumin–hyaluronan hydrogel showed promising results regarding metabolic disc cell activity and implant distribution in vivo and in vitro [27]. 

Unfortunately, potential risks, like extrusion, migration, and endplate changes, are common problems with devices or gels implanted into the nucleus pulposus [28,29,30,31]. Even moderate motions with low loads may provoke extrusion if the annulus is not intact due to a disc herniation or a nucleotomy, as shown in biomechanical experiments with tissue-engineered collagen-based gel implants. Even gluing or suturing the defect in the annulus does not seem to be a reliable solution (46). However, secure annulus fibrosus closure techniques may provide nucleus replacement approaches with a better chance [32]. An important step towards obtaining a successful solution is to minimize the defect size before hydrogel application. An advantage of in situ solidifying gels is that they can be injected as a rather fluid material, which can easily penetrate into the tissue. The subsequent solidification of the gel embeds it into the tissue environment. Therefore, the present study suggests that the injection of the gel through the intact annulus using a syringe with a small needle, followed by in situ solidification, may increase the chances of the gel staying inside the injection site—even if the disc is exposed to many physiological motions and heavy loads.

The objectives of this in vitro study were to evaluate the biomechanical behavior in terms of disc height, range of motion, extrusion risk, and the local retentive capacity of the two in situ solidifying gels after injection through an intact annulus fibrosus into the nucleus pulposus during cyclic physiological biomechanical testing. 

## 2. Results and Discussion 

### 2.1. Injection Volume

The injection volume in the gelatine group ranged between 0.27 and 0.53 mL (mean value: 0.39 mL), and the injection volume in the albumin group ranged between 0.35 mL and 0.72 mL (mean value: 0.48 mL). The recommended injection time of between 30 and 90 s could be achieved in all specimens for both gel groups.

### 2.2. Height Change

**Figure 1:**
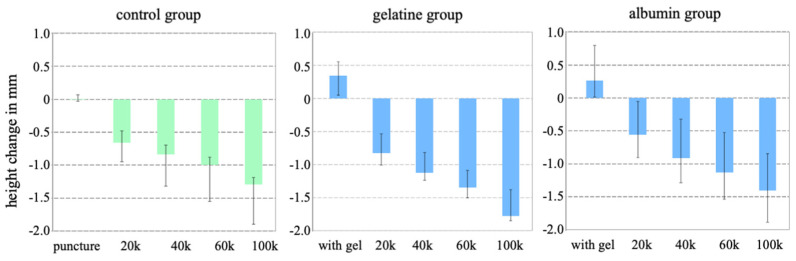
Median values (min–max) of height change in mm after treatment (needle puncture in the control group and gel injection in the gel groups) and after each cyclic loading bloc (e.g., 20k = 20,000 cycles) in relation to the intact state (zero position) of each test group.

After the injection of the gels, the height of the specimens in the gelatine group increased, with a median value of 0.34 mm (0.05–0.56 mm), and the height of the specimens increased in the albumin group with a median value of 0.27 mm (0.01–0.80 mm). In contrast, in the control group, needle puncture alone did not lead to a height change (Figure 1). 

During dynamic loading, the height decreased gradually in all specimens, independent of the test group. The hight loss for the albumin gel group was 1.40 mm (0.84–1.89 mm) and was similar for the control group, which received needle puncture alone. The height loss in the gelatine group was 1.78 mm (1.38–1.85 mm).

### 2.3. Range of Motion

All three test groups were comparable in the intact state and did not show significant differences in range of motion (ROM) (Table 1). 

**Figure 2:**
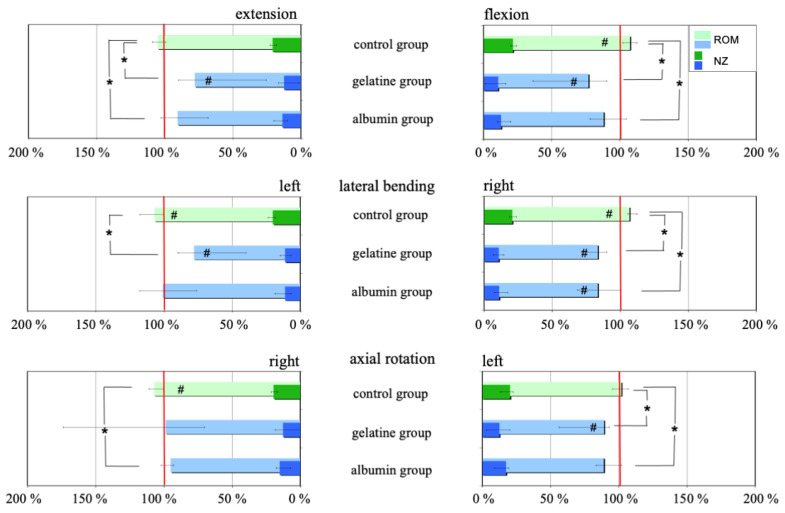
ROM and NZ after treatment (needle puncture in the control group and gel injection in the gel groups) of each group (n = 6), which were normalized to the respective intact state (100%, red line). # shows significant differences between intact condition and after first treatment within each group (*p* < 0.05; Wilcoxon Signed Rank test). * shows significant differences between control group and gel groups (*p* < 0.05; Wilcoxon Mann–Whitney test).

Gel injection decreased the ROM in both implant groups, with a larger decrease observed for the gelatine group than for the albumin group (Figure 2 and Table 1). The needle puncture alone received by the control group increased the ROM only slightly in all motion planes.

During cyclic loading, with a maximum of 100,000 cycles, the ROM continuously increased in all test groups (Table 1). 

### 2.4. Extrusion Risk

In both gel groups, no extrusion of the injected gels was observed. A small droplet was only visible on the outside one specimen of the albumin gel group after the removal of the injection needle. However, this droplet was so small that its weight could not be quantified, even with a precision balance. The weight of this droplet was less than 0.1 mL, corresponding to less than 2% of the average volume of the injected gel. Thus, it can be inferred that the leaked material does not originate from the nucleus itself, but rather emanates from the injection channel.

### 2.5. Macroscopic Examination

**Figure 3:**
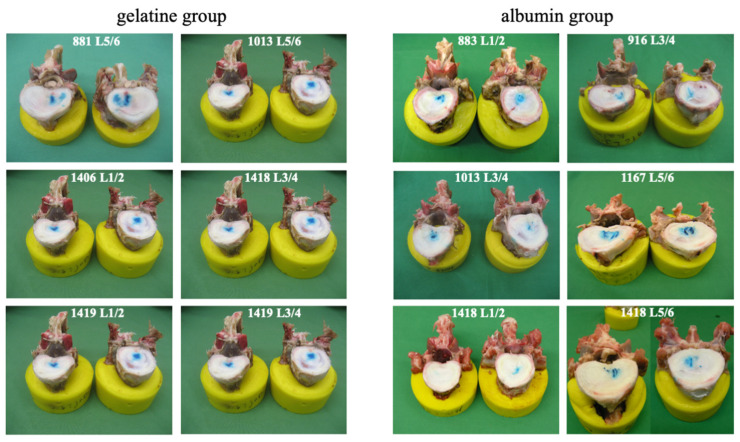
Disc cut in the transversal plane shows the gel distribution of the two gels, pigmented blue for better visualization, in the implanted bovine specimens after end of the dynamic loading (100,000 cycles).

After conducting biomechanical testing, the macroscopic inspection of the transverse sections revealed an accumulation of polymerized gel at the end of the injection canal within all specimens of the gel groups. The polymerized gel was securely adhered to the nucleus material (Figure 3).

### 2.6. Discussion

In the present study, two different in situ solidifying gels, serving as carrier materials for autologous-disc-derived chondrocyte transplantation (ADCT), were injected into an intervertebral disc and subjected to cyclical loading involving complex motions. Approximately 0.3 mL of the gel was successfully injected, resulting in a slight increase in disc height and motion segment stiffness. Cyclic loading with 100,000 cycles decreased these biomechanical restorations. The key finding was that no extrusion was observed, indicating that injecting gels through an intact annulus fibrosus holds promise as a viable solution for the therapeutic application of gels in disc regeneration. 

Both hydrogels in this study were chosen due to their suitability for the cultivation of different chondrogenic cell types, with high viability and high levels of differentiation. HA was added to the gels as a hydrodynamic component at an equivalent concentration to the concentration in the healthy disc (approximately 0.3–0.4%) [33]. HA is already used in the treatment of degenerative joint diseases. The anti-inflammatory and cell-protective properties of HA might have a beneficial influence on the success of a cell therapy [34].

In situ solidifying hydrogels have the advantage that they can be applied arthroscopically, causing only minimal damage [20]. This minimally invasive approach is suitable for locations that are difficult to access, such as the IVD. These gels show an excellent integration into the native tissue because they are injected as a liquid; therefore, they can penetrate into all tissue cavities prior to in situ solidification. The solidified hydrogels are soft, with a viscosity between a gel-like and solid-like form. 

Albumin hyaluronic acid gel has been clinically used for many years for the transplantation of autologous chondrocytes for the treatment of large cartilage defects. A clinical trial showed clinically meaningful improvements in up to 2 years’ follow-up [35].

The transverse cuts showed that, after repeated loading cycles, the gels stayed at the injection site in the center of the nucleus pulposus, with a close connection and retention to the surrounding tissue environment (Figure 3). The potential combination of this in situ solidifying gel and cells has the advantage of keeping them at the injection site. The gels have further beneficial retentive properties towards newly synthesized ECM components, keeping them in the vicinity of the cells. This suggests that combining the gels with the cells is superior to the injection of a fluid suspension for which extrusion was reported [15,16]. 

The disc height was shown to slightly increase with the gel injection, although this increase was quickly lost following cyclical loading. At the same time, the flexibility (in terms of ROM) increased between ca. 25% and 35% depending on the motion plane; the felxibility was strongest in flexion-extension and weakest in axial rotation. This can be explained by the viscoelastic behavior of the annuls and the other soft tissues, and is probably predominantly due to the fluid that is continuously pressed out of the disc with this complex loading over a total period of 6–8 h. This also explains why both the decrease in height and increase in ROM are similar for all three groups, whether with gels or without gel. Both changes are comparable to those observed in previous experiments [36].

In this study, bovine functional spinal units were used because they have been shown to be an adequate model for such investigations. It has been been shown that calf spines are a valid model for biomechanical flexibility testing [37]. They also have similar anatomical dimensions [38]. Human specimens are more expensive and hard to obtain, and the conditions of the individual specimens can be very different in terms of degeneration. We used specimens from 5–6-month-old calves, which usually show no signs of degeneration and thus provide comparable conditions. Furthermore, they have an intact and non-degenerated nucleus, which guarantees a higher level of intradiscal pressure, resulting in a worst-case scenario for the testing of the polymerized gel.

This complex multi-axial dynamic loading situation was simulated with bending moments of 24 Nm. Although this loading was within the physiological range, it represents the worst-case situation [39,40,41,42]. The dynamic cyclic test protocol that was used has already been proven, in various biomechanical studies, to provoke disc herniation or the extrusion of nucleus implants [43], and can be used to test annuls closure devices [44] and to compare vertebroplasty and kyphoplasty [36]. 

The number of loading cycles was limited to 100,000 to avoid the autolysis of the specimens [45]. Nevertheless, the number of cycles represented the loading within the first few post-operative weeks or months. The whole test procedure for one specimen, including thawing, preparation, flexibility tests, implant injection, height measurements, and cyclic loading, was performed within 20 h, since biomechanical property changes could be neglected for the duration of this test.

A limitation of the present study is the use of a hydrogel without cell application, as cell-seeded hydrogels may change their biomechanical properties due to the remodeling of the scaffold, de novo matrix synthesis, or matrix degradation. This means that long-term application in high-quality comparative in vivo approaches is required to investigate how physiological loading influences the safety and efficacy of such a treatment over a longer application period.

## 3. Conclusions

Overall, the results suggest that the injection of in situ solidifying gels has the potential to anchor autologous-disc-derived chondrocytes, serving as a source of regenerative cell populations, into the damaged nucleus pulposus. If they are injected through the intact annulus using a thin needle, they have a much lower risk of being extruded compared to most other solutions and might provide the regenerative strategies using cells with a better chance of success.

## 4. Materials and Methods

### 4.1. Implants

In the present study, the application of two different in situ solidifying gels, a gelatine gel and an albumin gel, serving as carrier materials for autologous-disc-derived chondrocyte transplantation (ADCT), were biomechanically tested under cyclic loading. Both gels were supplemented with 0.4% hyaluronic acid as a hydrodynamic additive [46]. The materials were developed and provided from TETEC AG and the Naural Science and Medical Sciences Institute (NMI) at the University of Tübingen, Tübingen, Germany. The raw materials used for the preparation of the gelatine gels (gelatine and transglutaminase) were kindly provided by Gelita AG (Ebersbach, Germany). Both test gels consisted of two components: a solution containing the hydrogel components, in which cells can be incorporated, and a crosslinking solution. By mixing the two components, the crosslinking reaction is started and the application gel is formed in situ in the target tissue. 

The transglutaminase crosslinked gelatine gel implant, short gelatine gel, consists of 10% gelatine from pig skin and 0.4% hyaluronic acid (Visiol, TRB Chemedica AG, Haar, Germany). The gelatine is enzymatically crosslinked through the addition of 0.15 U transglutaminase. 

The suspension was applied with a double chamber syringe: one chamber contained the suspension of the hydrogel components and one chamber included the crosslinker and a mixing device. A scale allowed for the application volume to be controlled. The time required for gelatine gel formation was approximately thirty minutes [47].

The albumin hyaluronic acid gel implant, short albumin gel, is based on a chemically modified albumin (43 mM maleimide groups) and 0.4% hyaluronic acid. The crosslinking solution comprised bis-thio polyethylene glycol (10,000 g/mol, 15 mM SH groups) in 0.1 mM HCl. As detailed above, both components were mixed (4:1 ratio) and applied with a double chamber syringe. The formation time of the albumin gel was approximately 3–5 min [26]. 

### 4.2. Specimens and Preparation

Six complete fresh-frozen lumbar (L1–L6) bovine specimens (Butchery Mutschler, Zainingen, Germany) of the age of 5–6 months were used [37]. The specimens were freshly dissected, wrapped in double plastic bags, and frozen at −20 °C. To exclude damaged spine specimens, lateral and postero-anterior X-rays were taken prior to testing. Before testing, the specimens were thawed overnight at 4 °C and prepared at room temperature. All soft tissue was removed, leaving the discs, ligaments, and joint capsules intact. Then, the specimens were divided into 18 functional spinal units (FSU) (L1–L2, L3–L4 and L5–L6).

The cranial respectively caudal half of the vertebral bodies was embedded in polymethylmethacrylate (PMMA, Technovit 3040, Heraeus Kulzer, Wehrheim, Germany) and flanges were mounted on the PMMA blocks to connect them to the different test apparatus. The FSUs were assigned to three test groups with n = 6 specimens (2 × L1–L2; 2 × L3–L4; 2 × L5–L6).

### 4.3. Treatments

The gelatine gel has to be injected at 37 °C body temperature to ensure sufficient polymerization. Therefore, the specimens were placed for eight hours at 37 °C in the incubator before starting the test. The gelatine gel and the cross-linker were warmed for 90 min to 40 °C. To prevent the specimens from drying, they were left in the plastic bags. An injection volume of 0.3–0.5 mL was intended, within an injection time of 90 s, to ensure that the temperature was still 37 °C during injection. The exact volume was detected by weighing the difference with a precision balance. During injection, the specimen was placed horizontally, the 18-gauge needle with a diameter of 1.2 mm (Terumo 1.2 × 50 mm, B.Braun, Melsungen, Germany) was inserted laterally from the right side, and the tip was positioned at the center of the nucleus. During the gelation time of 30 min, the specimen, together with the double-chamber syringe in the disc, were again placed in the incubator at 37 °C. To prevent the specimens from drying during this process, they were wrapped with gauze-soaked saline solution (0.9%), and additionally with cling wrap. After polymerization, the double-chamber syringe was removed.

The albumin gel was injected at room temperature. Again, the intention was to inject as much as possible, but at least a volume 0.3–0.5 mL, within an injection time of 90 s. The injection was carried out as described above. The albumin gel was formed at room temperature and, after the three minutes of gel formation, the double-chamber syringe with an 18-gauge needle was removed. 

A third group, with no gel, but with an 18-gauge needle puncture in the right lateral side, served as the control group. 

### 4.4. Height Measurements

**Figure 4:**
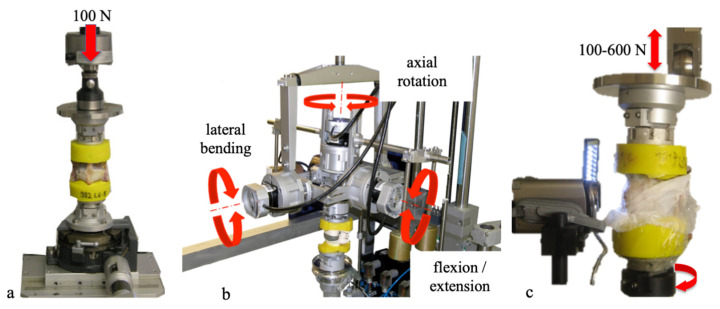
Height measurement (**a**), flexibility tests (**b**), and dynamic cyclic loading (**c**).

Before each flexibility test, the height of the motion segment was measured using a dynamic material testing machine (Instron 8871, Instron Wolpert GmbH, Darmstadt, Germany) under standardized conditions, with a centric static axial preload of 100 N applied using a flat piston (Figure 4a). 

### 4.5. Flexibility Tests

The biomechanical characteristics of the untreated and treated segment, as well as the segment after 20,000, 40,000, 60,000, and 100,000 dynamic loading cycles, were determined with flexibility tests using a spine tester (Figure 4b) [48].

As recommended for lumbar spinal implant testing pure moments of ±7.5 Nm were applied in lateral bending right/left, flexion/extension, and axial rotation left/right without preload [49,50]. The three-dimensional rotational movements of the segment were recorded using rotary variable displacement transducers (P2701A502, Novotechnik, Ostfildern, Germany; resolution 0.1°), which were integrated into the spine tester. During loading, the specimen could move, unconstrained, in the five uncontrolled degrees of freedom. The bending moments and the resulting rotations of the specimen were recorded continuously. To minimize the viscoelastic effects of the specimen, two loading cycles were applied for preconditioning, while the third cycle was used to evaluate the range of motion (ROM) and neutral zone (NZ). To allow for a better comparison within each group after treatment (with the needle puncture in the control group and after the gel injection in the implant groups), the results were normalized to the individual intact state (100%).

All specimens were kept moist with saline solution to avoid dehydration during testing [45].

### 4.6. Dynamic Cyclic Loading

The dynamic testing protocol was adapted from Wilke et al. to expose the specimens to up to 100,000 load and motion cycles to provoke extrusion (Figure 4c) [36,43]. 

The specimens were fixed via the caudal vertebrae onto a custom-built rotation platform mounted on the dynamic material testing machine. An axial sinusoidal load of 5 Hz, with a minimum of 100 N and a maximum of 600 N, was applied using a lever arm of 40 mm to the upper plate, mounted on the cranial vertebrae of the specimen. This resulted in an alternating bending moment between 4 and 24 Nm. During this dynamic loading, the set-up rotated clockwise at 360°/min, starting in flexion and gradually passing with a right lateral bending, followed by an extension, and then left lateral bending, before starting the flexion again, etc. 

In order to document the possible gel extrusion during dynamic testing, a digital video camera (JVC Everio, JVC Deutschland GmbH, Friedberg, Germany) was fixed to the rotating platform on the gel injection side.

### 4.7. Macroscopic Inspection

For macroscopic inspection and the final evaluation, the specimens were cut transversally at the level of the injection canal. The central injection into the disc was assessed and the gel distribution was inspected.

### 4.8. Data Analysis

Since only n = 6 specimens per group were tested, normal distribution of the data cannot be assumed; therefore, median values with ranges are presented. 

For statistical analysis, the Kruskal–Wallis test was employed to ascertain whether significant differences existed among the three test groups in the intact condition.

To compare the intact state with the state after treatment within each test group, the non-parametric Wilcoxon Signed Rank test was utilized.

To investigate whether there are significant differences between the control group and each gel group after treatment, the Wilcoxon Mann–Whitney test was used. Therefore, the normalized values were used. 

A significance level of 0.05 was set for all tests.

## Figures and Tables

**Table 1:** Total ROM in ° with median values (min–max) for the three principal motion planes at ±7.5 Nm, intact, after treatment, and after cyclic loading (c. = cycles).

Flexion/ Extension	Intact	After Treatment	After 20,000 c.	After 40,000 c.	After 60,000 c.	After 100,000 c.
Control	5.7 (4.7–11.2)	6.2 (4.8–11.7)	8.1 (6.9–13.6)	8.7 (7.5–14.1)	9.6 (7.8–14.5)	10.0 (8.6–14.5)
Albumin	6.4 (5.7–15.0)	5.8 (5.1–11.0)	8.1 (6.6–14.9)	9.4 (7.5–16.2)	9.8 (7.9–17.6)	10.6 (8.6–18.5)
Gelatine	6.1 (5.1–16.2)	4.9 (3.9–7.4)	6.9 (5.0–11.3)	7.9 (6.3–12.5)	8.4 (6.2–13.8)	9.5 (6.4–14.8)
Lateral bending	Intact	After treatment	After 20,000 c.	After 40,000 c.	After 60,000 c.	After 100,000 c.
Control	11.2 (6.8–12.1)	12.0 (7.3–12.9)	14.4 (9.3–17.4)	15.2 (9.7–18.0)	15.7 (9.8–18.6)	16.0 (10.5–19.6)
Albumin	9.8 (7.1–12.0)	8.5 (6.2–12.5)	10.8 (9.2–16.1)	12.1 (10.1–17.7)	12.9 (10.6–18.7)	13.4 (10.9–20.5)
Gelatine	10.0 (7.8–17.4)	8.4 (6.3–10.4)	11.4 (8.1–13.6)	12.7 (9.4–14.8)	13.3 (10.0–15.5)	14.2 (10.6–17.2)
Axial rotation	Intact	After treatment	After 20,000 c.	After 40,000 c.	After 60,000 c.	After 100,000 c.
Control	4.1 (3.4–5.3)	4.2 (3.6–5.5)	5.4 (4.1–6.3)	5.5 (4.1–6.7)	5.5 (4.2–7.1)	5.9 (4.4–7.1)
Albumin	4.3 (3.4–6.5)	4.2 (3.0–5.8)	5.2 (3.3–6.8)	5.6 (3.9–7.1)	5.8 (4.3–7.3)	6.0 (4.0–7.8)
Gelatine	4.0 (3.0–4.8)	3.3 (2.9–4.4)	4.3 (3.0–5.7)	4.4 (3.5–6.0)	4.6 (3.2–6.2)	4.7 (3.4–6.7)

## Data Availability

All data are stored at the Institute of Orthopaedic Research and Biomechanics, Centre of Trauma Research Ulm, Ulm University, Germany.

## References

[1] Balkovec C., Vernengo J., McGill S.M. (2013). The use of a novel injectable hydrogel nucleus pulposus replacement in restoring the mechanical properties of cyclically fatigued porcine intervertebral discs. J. Biomech. Eng..

[2] Berlemann U., Schwarzenbach O. (2009). An injectable nucleus replacement as an adjunct to microdiscectomy: 2 year follow-up in a pilot clinical study. Eur. Spine J..

[3] Hegewald A.A., Knecht S., Baumgartner D., Gerber H., Endres M., Kaps C., Stussi E., Thome C. (2009). Biomechanical testing of a polymer-based biomaterial for the restoration of spinal stability after nucleotomy. J. Orthop. Surg. Res..

[4] Malhotra N.R., Han W.M., Beckstein J., Cloyd J., Chen W., Elliott D.M. (2012). An injectable nucleus pulposus implant restores compressive range of motion in the ovine disc. Spine (Phila Pa 1976).

[5] Meisel H.J., Ganey T., Hutton W.C., Libera J., Minkus Y., Alasevic O. (2006). Clinical experience in cell-based therapeutics: Intervention and outcome. Eur. Spine J..

[6] Meisel H.J., Siodla V., Ganey T., Minkus Y., Hutton W.C., Alasevic O.J. (2007). Clinical experience in cell-based therapeutics: Disc chondrocyte transplantation A treatment for degenerated or damaged intervertebral disc. Biomol. Eng..

[7] Ruan D.K., Xin H., Zhang C., Wang C., Xu C., Li C., He Q. (2010). Experimental intervertebral disc regeneration with tissue-engineered composite in a canine model. Tissue Eng. Part A.

[8] Sivan S.S., Roberts S., Urban J.P., Menage J., Bramhill J., Campbell D., Franklin V.J., Lydon F., Merkher Y., Maroudas A. (2014). Injectable hydrogels with high fixed charge density and swelling pressure for nucleus pulposus repair: Biomimetic glycosaminoglycan analogues. Acta Biomater..

[9] Varma D.M., Lin H.A., Long R.G., Gold G.T., Hecht A.C., Iatridis J.C., Nicoll S.B. (2018). Thermoresponsive, redox-polymerized cellulosic hydrogels undergo in situ gelation and restore intervertebral disc biomechanics post discectomy. Eur. Cell Mater..

[10] Wilke H.J., Heuer F., Neidlinger-Wilke C., Claes L. (2006). Is a collagen scaffold for a tissue engineered nucleus replacement capable of restoring disc height and stability in an animal model?. Eur. Spine J..

[11] Reitmaier S., Kreja L., Gruchenberg K., Kanter B., Silva-Correia J., Oliveira J.M., Reis R.L., Perugini V., Santin M., Ignatius A. (2014). In vivo biofunctional evaluation of hydrogels for disc regeneration. Eur. Spine J..

[12] Schmitz T.C., Salzer E., Crispim J.F., Fabra G.T., LeVisage C., Pandit A., Tryfonidou M., Maitre C.L., Ito K. (2020). Characterization of biomaterials intended for use in the nucleus pulposus of degenerated intervertebral discs. Acta Biomater..

[13] Mern D.S., Walsen T., Beierfuss A., Thome C. (2021). Animal models of regenerative medicine for biological treatment approaches of degenerative disc diseases. Exp. Biol. Med. (Maywood).

[14] Meisel H.J., Agarwal N., Hsieh P.C., Skelly A., Park J.B., Brodke D., Wang J.C., Yoon S.T., Buser Z. (2019). Cell Therapy for Treatment of Intervertebral Disc Degeneration: A Systematic Review. Glob. Spine J..

[15] Omlor G.W., Bertram H., Kleinschmidt K., Fischer J., Brohm K., Guehring T., Anton M., Richter W. (2010). Methods to monitor distribution and metabolic activity of mesenchymal stem cells following in vivo injection into nucleotomized porcine intervertebral discs. Eur. Spine J..

[16] Bertram H., Kroeber M., Wang H., Unglaub F., Guehring T., Carstens C., Richter W. (2005). Matrix-assisted cell transfer for intervertebral disc cell therapy. Biochem. Biophys. Res. Commun..

[17] Loibl M., Wuertz-Kozak K., Vadala G., Lang S., Fairbank J., Urban J.P. (2019). Controversies in regenerative medicine: Should intervertebral disc degeneration be treated with mesenchymal stem cells?. JOR Spine.

[18] Buckley C.T., Hoyland J.A., Fujii K., Pandit A., Iatridis J.C., Grad S. (2018). Critical aspects and challenges for intervertebral disc repair and regeneration-Harnessing advances in tissue engineering. JOR Spine.

[19] Pereira D.R., Silva-Correia J., Oliveira J.M., Reis R.L. (2013). Hydrogels in acellular and cellular strategies for intervertebral disc regeneration. J. Tissue Eng. Regen. Med..

[20] Bowles R.D., Setton L.A. (2017). Biomaterials for intervertebral disc regeneration and repair. Biomaterials.

[21] Reza A.T., Nicoll S.B. (2010). Characterization of novel photocrosslinked carboxymethylcellulose hydrogels for encapsulation of nucleus pulposus cells. Acta Biomater..

[22] Yan C., Wang X., Xiang C., Wang Y., Pu C., Chen L., Jiang K., Li Y. (2021). Applications of Functionalized Hydrogels in the Regeneration of the Intervertebral Disc. Biomed. Res. Int..

[23] Zhang Y.S., Khademhosseini A. (2017). Advances in engineering hydrogels. Science.

[24] Peroglio M., Eglin D., Benneker L.M., Alini M., Grad S. (2013). Thermoreversible hyaluronan-based hydrogel supports in vitro and ex vivo disc-like differentiation of human mesenchymal stem cells. Spine J..

[25] Peroglio M., Grad S., Mortisen D., Sprecher C.M., Illien-Junger S., Alini M., Eglin D. (2012). Injectable thermoreversible hyaluronan-based hydrogels for nucleus pulposus cell encapsulation. Eur. Spine J..

[26] Benz K., Stippich C., Osswald C., Gaissmaier C., Lembert N., Badke A., Steck E., Aicher W.K., Mollenhauer J.A. (2012). Rheological and biological properties of a hydrogel support for cells intended for intervertebral disc repair. BMC Musculoskelet. Disord..

[27] Omlor G.W., Fischer J., Kleinschmitt K., Benz K., Holschbach J., Brohm K., Anton M., Guehring T., Richter W. (2014). Short-term follow-up of disc cell therapy in a porcine nucleotomy model with an albumin-hyaluronan hydrogel: In vivo and in vitro results of metabolic disc cell activity and implant distribution. Eur. Spine J..

[28] Pimenta L., Marchi L., Coutinho E., Oliveira L. (2012). Lessons Learned After 9 Years’ Clinical Experience with 3 Different Nucleus Replacement Devices. Semin. Spine Surg..

[29] Klara P.M., Ray C.D. (2002). Artificial nucleus replacement: Clinical experience. Spine (Phila Pa 1976).

[30] Di Martino A., Vaccaro A.R., Lee J.Y., Denaro V., Lim M.R. (2005). Nucleus pulposus replacement: Basic science and indications for clinical use. Spine (Phila Pa 1976).

[31] Bao Q.B., McCullen G.M., Higham P.A., Dumbleton J.H., Yuan H.A. (1996). The artificial disc: Theory, design and materials. Biomaterials.

[32] Zengerle L., Kohler A., Debout E., Hackenbroch C., Wilke H.J. (2020). Nucleus replacement could get a new chance with annulus closure. Eur. Spine J..

[33] Scott D., Coleman P.J., Mason R.M., Levick J.R. (2000). Concentration dependence of interstitial flow buffering by hyaluronan in synovial joints. Microvasc. Res..

[34] Presti D., Scott J.E. (1994). Hyaluronan-mediated protective effect against cell damage caused by enzymatically produced hydroxyl (OH.) radicals is dependent on hyaluronan molecular mass. Cell Biochem. Funct..

[35] Niemeyer P., Hanus M., Belickas J., Laszlo T., Gudas R., Fiodorovas M., Cebatorius A., Pastucha M., Hoza P., Magos K. (2022). Treatment of Large Cartilage Defects in the Knee by Hydrogel-Based Autologous Chondrocyte Implantation: Two-Year Results of a Prospective, Multicenter, Single-Arm Phase III Trial. Cartilage.

[36] Wilke H.J., Mehnert U., Claes L.E., Bierschneider M.M., Jaksche H., Boszczyk B.M. (2006). Biomechanical evaluation of vertebroplasty and kyphoplasty with polymethyl methacrylate or calcium phosphate cement under cyclic loading. Spine (Phila Pa 1976).

[37] Wilke H.J., Krischak S.T., Wenger K.H., Claes L.E. (1997). Load-displacement properties of the thoracolumbar calf spine: Experimental results and comparison to known human data. Eur. Spine J..

[38] Cotterill P.C., Kostuik J.P., D’Angelo G., Fernie G.R., Maki B.E. (1986). An anatomical comparison of the human and bovine thoracolumbar spine. J. Orthop. Res..

[39] Adams M.A. (1995). Mechanical testing of the spine. An appraisal of methodology, results, and conclusions. Spine (Phila Pa 1976).

[40] Rohlmann A., Bergmann G., Graichen F. (1997). Loads on an internal spinal fixation device during walking. J. Biomech..

[41] Rohlmann A., Bergmann G., Graichen F. (1999). Loads on internal spinal fixators measured in different body positions. Eur. Spine J..

[42] Rohlmann A., Graichen F., Weber U., Bergmann G. (2000). 2000 Volvo Award winner in biomechanical studies: Monitoring in vivo implant loads with a telemeterized internal spinal fixation device. Spine (Phila Pa 1976).

[43] Wilke H.J., Ressel L., Heuer F., Graf N., Rath S. (2013). Can prevention of a reherniation be investigated? Establishment of a herniation model and experiments with an anular closure device. Spine (Phila Pa 1976).

[44] Heuer F., Ulrich S., Claes L., Wilke H.J. (2008). Biomechanical evaluation of conventional anulus fibrosus closure methods required for nucleus replacement. Laboratory investigation. J. Neurosurg. Spine.

[45] Wilke H.J., Jungkunz B., Wenger K., Claes L.E. (1998). Spinal segment range of motion as a function of in vitro test conditions: Effects of exposure period, accumulated cycles, angular-deformation rate, and moisture condition. Anat. Rec..

[46] Comper W.D., Zamparo O. (1990). Hydrodynamic properties of connective-tissue polysaccharides. Biochem. J..

[47] Muller J., Benz K., Ahlers M., Gaissmaier C., Mollenhauer J. (2011). Hypoxic conditions during expansion culture prime human mesenchymal stromal precursor cells for chondrogenic differentiation in three-dimensional cultures. Cell Transpl..

[48] Wilke H.J., Claes L., Schmitt H., Wolf S. (1994). A universal spine tester for in vitro experiments with muscle force simulation. Eur. Spine J..

[49] Wilke H.J., Rohlmann A., Neller S., Schultheiss M., Bergmann G., Graichen F., Claes L.E. (2001). Is it possible to simulate physiologic loading conditions by applying pure moments? A comparison of in vivo and in vitro load components in an internal fixator. Spine (Phila Pa 1976).

[50] Wilke H.J., Wenger K., Claes L. (1998). Testing criteria for spinal implants: Recommendations for the standardization of in vitro stability testing of spinal implants. Eur. Spine J..

